# Solid Pseudopapillary Neoplasm of the Pancreatic Tail With Splenic Hilar Involvement: A Case Report

**DOI:** 10.7759/cureus.117340

**Published:** 2026-10-01

**Authors:** Kumaresh Pandian, Surya Priya, Arya Sivakumaran, Manish Marlecha, Surendran Paramsivam

**Affiliations:** 1 General Surgery, Sri Ramachandra Institute of Higher Education and Research, Chennai, IND

**Keywords:** case report, distal pancreatectomy, pancreatic tumour, solid pseudopapillary neoplasm, spen, splenectomy

## Abstract

Solid pseudopapillary epithelial neoplasm (SPEN) is a rare pancreatic epithelial tumour that predominantly affects young women and is characterised by low malignant potential and favourable prognosis following complete surgical resection.

A 28-year-old woman presented with intermittent left upper abdominal pain and loss of appetite for one month. Physical examination and laboratory investigations, including tumour markers, were unremarkable. Contrast-enhanced computed tomography revealed a 7.4 × 7.0 × 6.4 cm well-defined cystic lesion with solid components arising from the pancreatic tail, suggestive of SPEN. The patient underwent distal pancreatectomy with splenectomy due to splenic hilar involvement. Histopathological examination and immunohistochemistry confirmed SPEN, demonstrating β-catenin and progesterone receptor positivity with a Ki-67 index <1%.

SPEN is an uncommon pancreatic tumour that typically presents with non-specific symptoms, often leading to delayed diagnosis. Characteristic imaging findings combined with histopathological and immunohistochemical evaluation are essential for accurate diagnosis. Complete surgical resection remains the treatment of choice and is associated with favourable outcomes.

SPEN should be considered in young women presenting with pancreatic cystic lesions and vague abdominal symptoms. Complete surgical excision by distal pancreatectomy with splenectomy can achieve excellent oncological outcomes, even in cases with splenic hilar involvement.

## Introduction

Solid pseudopapillary epithelial neoplasm (SPEN) is a rare and distinct pancreatic neoplasm characterised by generally indolent biological behaviour and a favourable prognosis following complete surgical resection [1]. It was first described by V. K. Frantz in 1959 and predominantly affects young women, particularly those in the second and third decades of life [2]. The true population-based incidence and prevalence of SPEN remain poorly defined because of its rarity. Available literature suggests that SPEN accounts for approximately 0.9-2.7% of exocrine pancreatic neoplasms and approximately 5% of cystic pancreatic neoplasms [3].

Here, we present a case of SPEN arising from the pancreatic tail in a young woman with direct macroscopic involvement of the splenic hilum and spleen, requiring distal pancreatectomy with en bloc splenectomy. This case highlights the diagnostic features of SPEN and the importance of individualising the surgical approach according to the extent of local tumour involvement.

## Case presentation

Patient information

A 28-year-old woman presented to the general surgery outpatient department with complaints of intermittent left-sided upper abdominal pain for a duration of one month, which was insidious in onset and gradually progressive and associated with a history of loss of appetite, non-radiating, with no specific aggravating or relieving factors. The patient's medical and surgical history was unremarkable, as she denied any prior significant illnesses or hospitalizations. The patient denied any history of alcohol or drug use, and reported no significant family history.

Physical examination

On clinical examination, the patient appeared to be in good general condition, with normal vital signs, including afebrile temperature, stable blood pressure, and heart rate within normal limits. The abdominal examination revealed a soft, non-tender, and non-distended abdomen, without any palpable masses or organomegaly.

Laboratory investigation

The patient's comprehensive metabolic panel, encompassing liver and renal function assessments, alongside a complete blood count, indicated values within the normal reference ranges. Additionally, the evaluation of tumour markers, such as CA 19-9 and carcinoembryonic antigen, did not reveal any abnormalities. Both serum amylase and lipase concentrations were also found to be within the acceptable normal range.

Imaging

Helical computed tomography (CT) with and without contrast revealed a relatively well-defined lobulated hypodense cystic lesion of size 7.4 × 7.0 × 6.4 cm with few ill-defined isodense solid components within arising from the tail of the pancreas demonstrating the “claw sign”. The contrast-enhanced imaging revealed a predominantly cystic lesion with peripheral wall enhancement. Within the cystic lesion, there was a relatively well-defined, rounded solid component measuring 4.9 × 4.5 × 3.8 cm, which demonstrated progressive heterogeneous centripetal enhancement with a central non-enhancing area as shown in Figure 1A-1B. These features were indicative of an SPEN.

**Figure 1:**
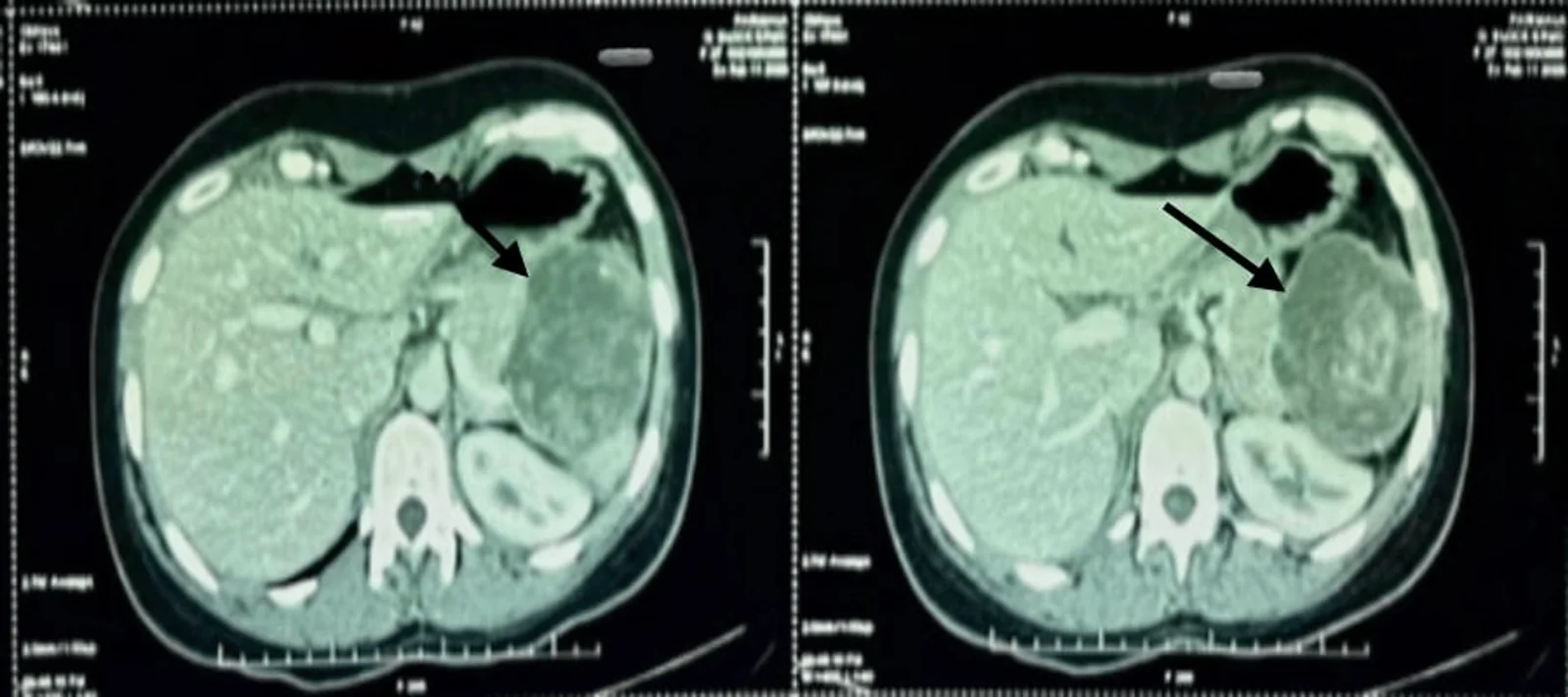
Contrast-enhanced computed tomography (CECT) whole abdomen axial view (A) and (B) shows a relatively well-defined lobulated cystic lesion of size 7.4 x 7.0 x 6.4 cm with ill-defined isodense solid components within arising from the tail of pancreas. The arrow indicates the lesion.

Differential diagnosis

In young patients presenting with a pancreatic tail lesion, the differential diagnosis includes solid pseudopapillary neoplasm, pancreatic neuroendocrine tumour, mucinous cystic neoplasm, serous cystic neoplasm, and, less commonly, pancreatic ductal adenocarcinoma. The differential diagnosis is guided by the patient's age, sex, clinical presentation, and characteristic radiological features. In the present case, the patient's young age, predominantly cystic lesion with a solid enhancing component, and subsequent histopathological and immunohistochemical findings supported the diagnosis of SPEN.

Treatment

Based on the patient's clinical presentation and characteristic radiological findings, SPEN of the pancreatic tail was suspected, and surgical resection was planned. Although laparoscopic and robotic distal pancreatectomy are established minimally invasive approaches for appropriately selected pancreatic tail lesions, an open approach was chosen in this patient because of the large tumour size and its macroscopic involvement of the splenic hilum and spleen. The open approach provided adequate exposure for safe dissection and en bloc resection of the involved structures.

Following multidisciplinary team discussion, the patient underwent distal pancreatectomy with splenectomy [4,5]. Intraoperative exploration revealed a tumour measuring approximately 9 × 8 × 7 cm arising from the pancreatic tail, with direct macroscopic invasion of the splenic hilum and involvement of the spleen, with displacement of the stomach anteriorly. Although spleen-preserving distal pancreatectomy is preferred when technically and oncologically feasible, preservation of the spleen was not considered feasible in this case because of direct tumour involvement of the splenic hilum and spleen. Therefore, splenectomy was performed en bloc with distal pancreatectomy solely because of the direct splenic hilar involvement, to permit complete resection of the involved structures and achieve an R0 resection.

Histopathological examination

The resected specimen consisted of a portion of distal pancreas and spleen (Figure 2). Gross examination revealed a well-circumscribed lesion in the tail of the pancreas with solid and cystic areas [6]. The cut surface revealed a solid cystic lesion measuring 7 × 6 × 5.5 cm, with both solid and cystic components. The pathological staging was pT3 N0. The tumour was found encroaching upon the splenic hilum, indicating involvement of the spleen's vascular structures.

**Figure 2:**
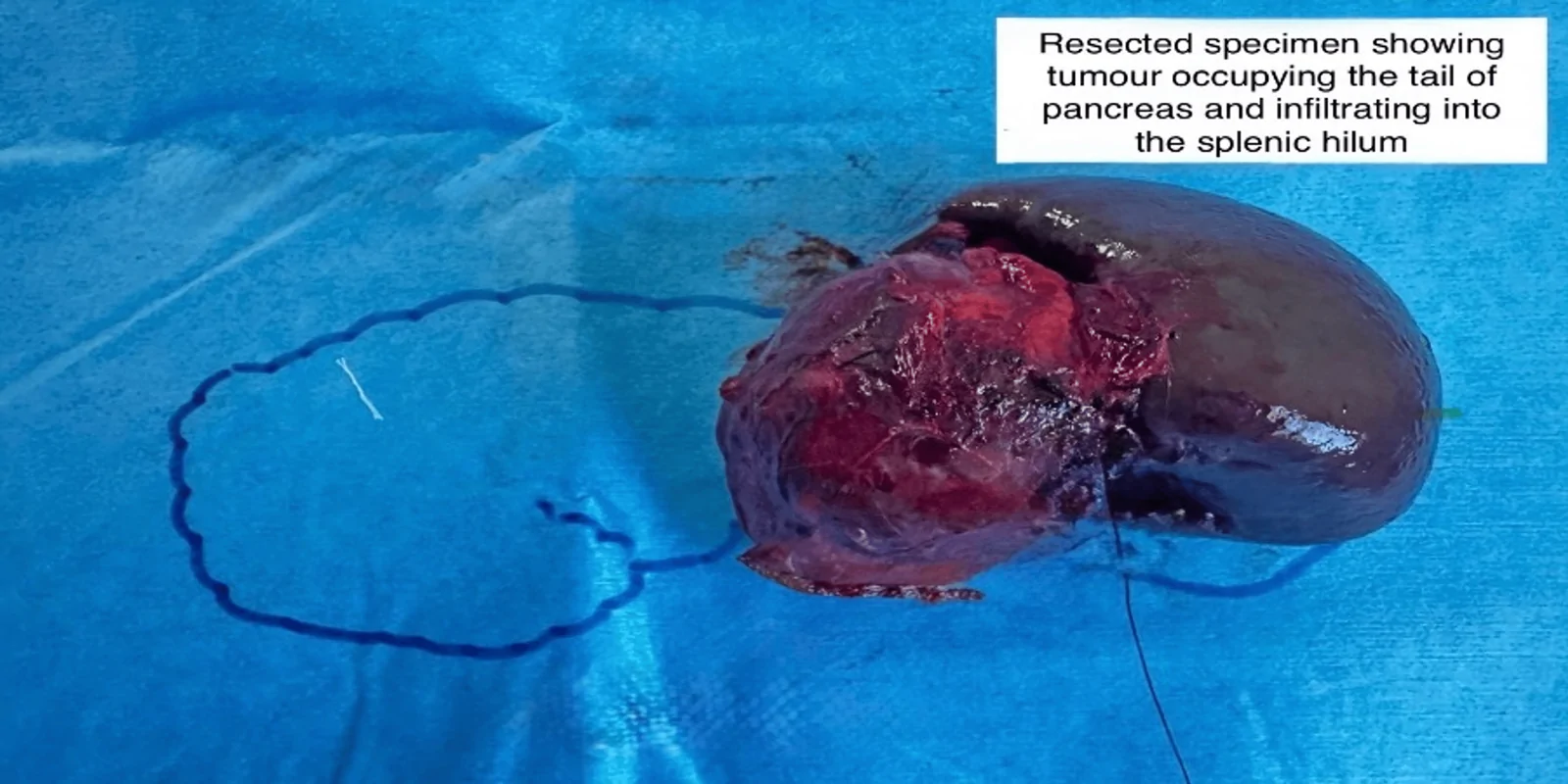
Resected tumour arising from the tail of the pancreas and infiltrating into the splenic hilum.

The microscopic analysis showed a solid pseudopapillary neoplasm, with the neoplastic cells organised in solid sheets, a pseudopapillary arrangement, and rosette formations [7]. The neoplastic cells exhibit moderate eosinophilic cytoplasm, an elevated nucleocytoplasmic ratio, dispersed chromatin, and inconspicuous nucleoli, as shown in Figure 3A-3B.

**Figure 3:**
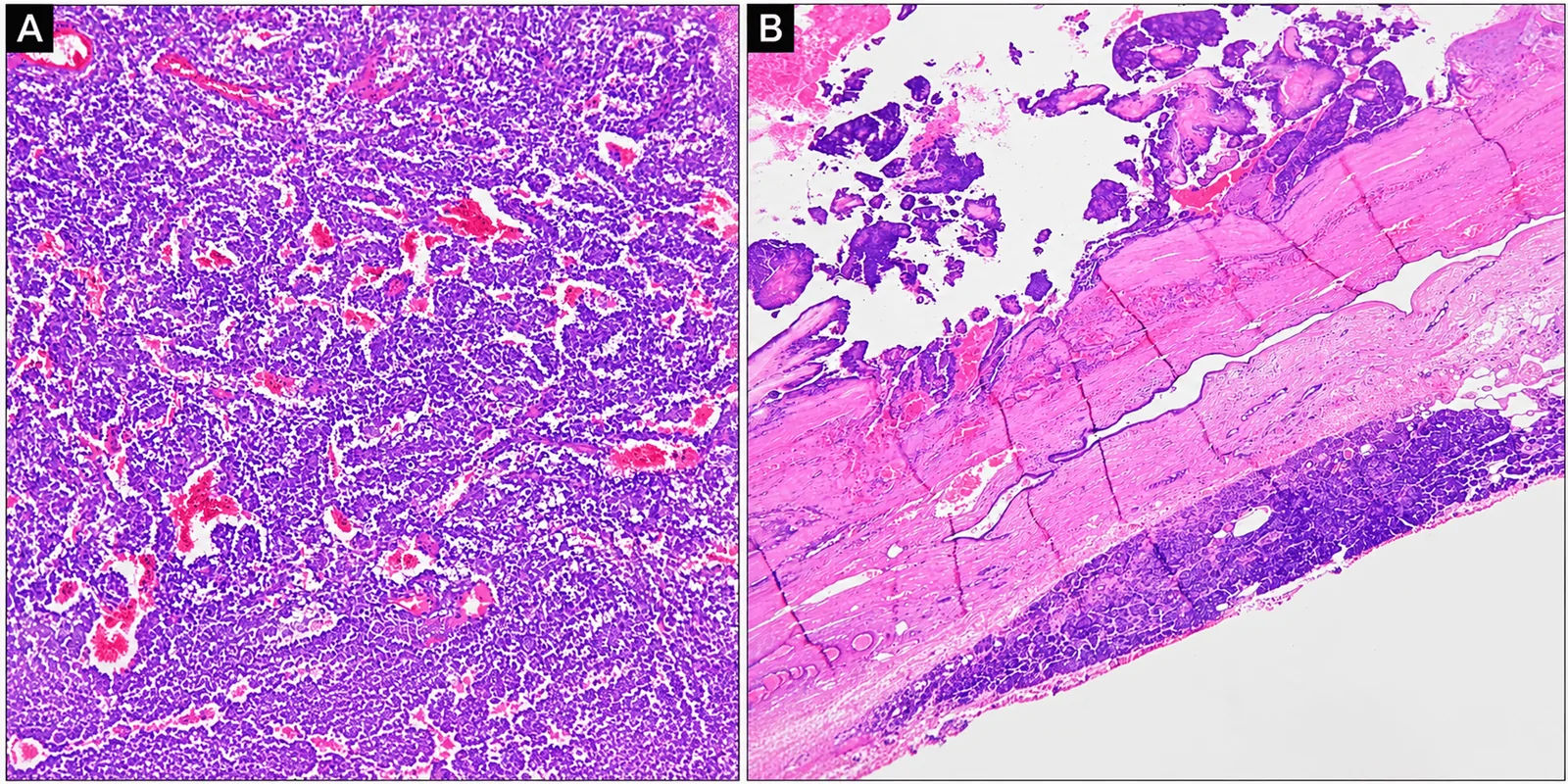
Histopathological features of solid pseudopapillary neoplasm of the pancreas. (A) High-power view section shows solid areas from neoplastic tissue with tumour cells arranged in papillary architecture (B) Low-power view section shows pancreatic parenchyma with underlying dense fibro-collagenous cyst wall lined by neoplastic cells arranged in papillary architecture

The immunohistochemical analysis found that the tumour cells were positive for beta-catenin and progesterone receptor, but negative for synaptophysin and chromogranin (Figure 4A-4D). Additionally, the proliferation rate, as measured by Ki-67 staining, was low at <1%, as shown in Figure 4E [8].

**Figure 4:**
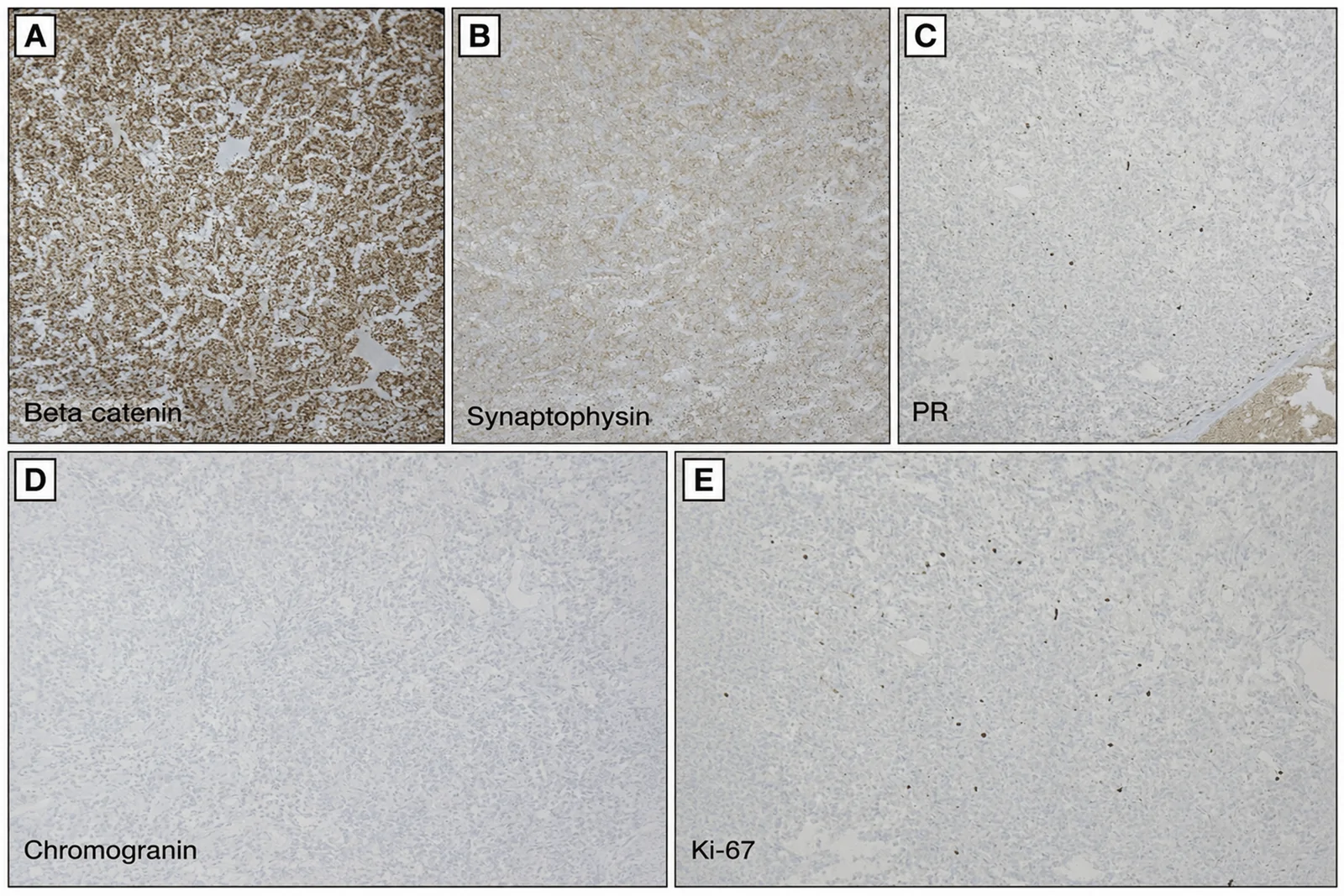
Immunohistochemical profile of solid pseudopapillary neoplasm of the pancreas (A) Beta catenin nuclear positivity (B) Synaptophysin negative (C) Progesterone receptor (PR) scattered nuclear positivity (D) Chromogranin negative (E) Ki67 <1%

Outcomes and follow-up

Our patient underwent distal pancreatectomy with splenectomy. Histopathological examination of the resected specimen confirmed the diagnosis of solid pseudopapillary neoplasm of the pancreas, with clear resection margins (R0 resection). The patient recovered well postoperatively and was discharged home on postoperative day seven. At the three-month follow-up, the patient remained asymptomatic, with no evidence of complications. A contrast-enhanced CT scan of the abdomen was performed, revealing no evidence of recurrence or distant metastasis. The patient is currently undergoing regular follow-up with clinical evaluation and cross-sectional imaging. Annual surveillance for at least five years following resection of solid pseudopapillary neoplasm is recommended by the American College of Gastroenterology Clinical Guideline, based on the potential risk of tumour recurrence. Given that late recurrences beyond five years have also been reported, continued long-term surveillance may be considered, particularly in patients with high-risk pathological features.

## Discussion

SPEN of the pancreas is a relatively infrequent yet intriguing form of pancreatic tumour, predominantly affecting young women, typically in their second to third decades of life. The presentation of our patient, in her late 20s, aligns statistically with the established demographic propensity for this neoplasm [9]. While SPENs are often discovered incidentally during imaging for unrelated conditions, symptomatic individuals may experience vague abdominal discomfort, nausea, or a palpable abdominal mass, depending on the tumour's size and location [10].

The exact etiology of SPENs remains elusive, but accumulating evidence points to genetic and molecular factors in its pathogenesis [11]. Specifically, mutations in the CTNNB1 gene, which encodes β-catenin, a crucial component of the Wnt signalling pathway, have been identified in a significant proportion of SPENs [12].

Histologically, SPENs are defined by a complex and unique cytoarchitecture, exhibiting both solid and pseudopapillary components, in addition to regions displaying cystic degeneration and haemorrhage [13]. Immunohistochemically, SPENs typically express a characteristic panel of markers, including β-catenin, vimentin, CD10, CD56, and α-1-antitrypsin. These markers aid in the differential diagnosis of SPENs from other pancreatic neoplasms.

The specific surgical approach is carefully determined based on several critical factors, including the precise anatomical location of the tumour, its overall size and extent of involvement of adjacent structures, and the presence or absence of distant metastasis. For tumours confined to the pancreatic tail, a distal pancreatectomy with or without splenectomy is typically performed, as was done in our patient, to ensure complete removal of the tumour while minimising the risk of recurrence [14]. When the neoplasm is situated in the pancreatic head or uncinate process, a pancreaticoduodenectomy, commonly known as the Whipple procedure, might be deemed necessary [15].

While surgical resection represents the primary treatment modality for SPENs, the role of adjuvant therapy in the management of these tumours remains controversial and is not routinely recommended [16]. Even in cases where the tumour exhibits persistent activity without significant changes in size or location, monitoring with imaging techniques like fluorodeoxyglucose-positron emission tomography (FDG-PET) can be valuable [17]. The incidental detection of neoplasms has risen significantly due to the increased use of advanced imaging technologies [18]. In some cases, surgeons, oncologists, and even pathologists may lack awareness of these rare tumours, resulting in diagnostic inaccuracies and delays [19]. Due to their rarity and diverse clinical and pathological characteristics, there is a pressing need for increased awareness, standardised diagnostic criteria, and collaborative research efforts to optimise the management of these tumours.

## Conclusions

In conclusion, this case underscores the efficacy of surgical resection as the primary treatment modality for SPEN. Our patient's favourable outcome following distal pancreatectomy with splenectomy reinforces the importance of complete tumour removal. Due to their rarity and diverse clinical and pathological characteristics, a high index of suspicion and awareness of SPEN are essential for timely diagnosis and appropriate management. Furthermore, given the potential for late recurrence, long-term surveillance with regular imaging is crucial to detect any disease recurrence early, ensuring optimal long-term outcomes for patients.
